# A review of traditional Chinese medicine intervention methods for depression among college students

**DOI:** 10.3389/fpsyg.2024.1506965

**Published:** 2025-01-31

**Authors:** Xiyong Yao, Chen Xu, Linlin Yang, Ailin Wu, Lin Xiong

**Affiliations:** ^1^College of Traditional Chinese Medicine, Chongqing Medical and Pharmaceutical College, Chongqing, China; ^2^Communication University of China Nanjing, Nanjing, China; ^3^School of Ethnology and Sociology, Yunnan University, Kunming, China; ^4^Chongqing Center for Disease Control and Prevention, Chongqing, China; ^5^College of Traditional Chinese Medicine, Chongqing Medical University, Chongqing, China

**Keywords:** TCM intervention for depression, depression in college students, TCM health preservation techniques, five-element music therapy, acupuncture treatment

## Abstract

Depression has become the most prevalent psychological issue among college students, necessitating urgent intervention measures. Traditional Chinese Medicine (TCM) boasts a long history in the prevention and treatment of psychological and emotional disorders, encompassing non-pharmacological therapies (such as TCM health exercises, Tai Chi, Wu Qin Xi, Ba Duan Jin, acupuncture, etc.) and pharmacological treatments. Key research findings indicate that these TCM interventions can significantly alleviate depressive symptoms and improve sleep quality in college students. However, the studies also acknowledge certain limitations, including varied effectiveness of interventions among individuals and the need for professional diagnosis and treatment in the case of acupuncture and herbal remedies. This provides early prevention and intervention measures for the treatment of depression among college students, and promotes the inheritance of traditional Chinese culture.

## 1 Introduction

Depression, also known as depressive mood, is a negative emotion and the most prevalent psychological issue among college students, serving as a major risk factor for suicidal ideation (He and Yang, 2015). The prevalence of depression among Chinese college students ranges from 15 to 40%, compared to just 5 to 6% in the general population, indicating a significantly higher and increasing rate (Song et al., 2020; Wang et al., 2020). If depression remains unaddressed and unimproved for a long time, it can develop into depressive disorder, a psychological disorder also known as major depressive disorder. The incidence of depressive disorder has been increasing globally year by year, and by 2020, it had become the second leading cause of death worldwide, after heart disease (Moussavi et al., 2007). Studies have shown that the prevalence of depression among Chinese college students is significantly higher than that reported in the general Chinese population (Li et al., 2016).This is because college students have certain unique characteristics that make them a high-risk group for depression. Factors such as their immature physical and mental development, interpersonal relationships, academic and employment pressures, social adaptation challenges, experiences of bullying, possible avoidant personality disorders (Xu et al., 2022), and external environmental influences like COVID-19 (Luo et al., 2021) contribute to their heightened susceptibility to depressive disorders (Shensa et al., 2017). How to effectively intervene in the depressive moods of college students at an early stage and prevent them from developing into depressive disorders is a topic that educators and medical professionals are constantly exploring. Studies have shown that psychological and psychiatric disorders such as depression and depressive disorders can be categorized under the scope of mental and emotional disorders in TCM, specifically under the category of “depressive syndromes.” TCM has a long history and rich experience in understanding, preventing, and treating mental and emotional disorders. For instance, the “Huangdi Neijing” (Yellow Emperor’s Classic of Internal Medicine) was the first to record the concept of the Five Elements’ depression and emotional factors leading to depression. “Lingshu: Benshen” (Spiritual Pivot: The Root of the Spirit) states, “Those who are worried and anxious have their qi obstructed and do not flow smoothly,” discussing how emotional imbalance can cause qi stagnation, leading to the onset of depressive syndromes. These syndromes are often caused by factors such as unresolved emotions of worry, contemplation, sadness, anger, excessive physical exertion, suppressed anger, unresolved thoughts, or unfulfilled desires, resulting in liver dysfunction, spleen deficiency, and heart malnutrition, leading to low mood. Traditional Chinese health exercises, which have been passed down for thousands of years, such as the combination of hardness and softness in Tai Chi, the unity of spirit and form in Wu Qin Xi (Five Animal Exercises), the stretching of body and mind in Ba Duan Jin (Eight Sections of Brocade), and the stretching of tendons and bones in Yi Jin Jing (Tendon-Changing Classic), not only fully stretch the meridians of college students’ bodies, regulate qi to nourish their organs, and enhance their vital energy, but also improve depressive symptoms by regulating their emotions, thereby contributing to the overall physical and mental health of college students.

This study primarily conducted searches in Chinese databases (CNKI, Wanfang, VIP) and English databases (PubMed, WOS, Embase, PsycInfo) using the keywords “college students’ depression” AND “health exercises” OR “Ba Duan Jin” OR “Tai Chi” OR “Five-Element Music” OR “five tones” OR “acupuncture” OR “Chinese herbal medicine.” Inclusion criteria: The study subjects were college students with depressive tendencies or symptoms; the experimental group received interventions using traditional Chinese medicine (TCM) methods; the experimental results discussed the intervention effects of TCM methods on college students’ depression. Exclusion criteria: Duplicate publications (as well as articles indexed in different databases but representing the same study), literature with unextractable outcome indicators or unrelated to the study’s indicators, and literature with study subjects other than college students were excluded. Finally, 29 typical studies were selected for the review. The aim is to provide research ideas and measures for researchers interested in exploring TCM methods for intervening in depression, hoping to offer valuable insights and approaches.

## 2 Non-medication TCM interventions

### 2.1 TCM health preservation techniques

Traditional Chinese health exercises, rooted in mindfulness-based aerobic therapy, encompass guided movements such as Tai Chi, Ba Duan Jin, Liu Zi Jue, Wu Qin Xi, Yi Jin Jing, and others. These exercises harmonize Yin and Yang and regulate the flow of Qi through coordinated breathing and physical movements (Li et al., 2022). Based on the principles of TCM, these exercises emphasize the integration of mind, Qi, body, and spirit, combining martial arts, guided movements, and breathing techniques. By regulating the body’s Qi and blood, unblocking meridians, nourishing organs, and balancing Yin and Yang, they achieve the goal of nurturing health and well-being (Yang et al., 2020).

Tai Chi is a traditional martial art form that not only includes martial arts techniques and strength training but also emphasizes a health-preserving philosophy integrated with TCM (Zhao et al., 2021). Ba Duan Jin is one of the traditional health exercises, consisting of eight different movements designed for fitness. Yi Jin Jing is an exercise method primarily aimed at strengthening the body and muscles. Its main characteristic is the combination of movement and stillness, with internal stillness used to calm the mind and regulate breathing, while external movement is used to strengthen the muscles and bones. Wuqinxi is a traditional Chinese fitness method invented by the famous doctor Hua Tuo in the late Eastern Han Dynasty, based on the movement patterns of five animals: tiger, deer, bear, monkey, and bird (or crane, according to some versions). It is officially recorded in the “Biography of Hua Tuo” in the “Book of the Later Han.”

Zhang Jingyi (Zhang et al., 2021) and others conducted a meta-analysis on the intervention effects of TCM health-preserving exercises such as Tai Chi, Baduanjin, Yijinjing, and Wuqinxi Qigong on improving negative psychological conditions such as anxiety and depression among college students. The study found that Tai Chi, Baduanjin, Yijinjing, and Wuqinxi can effectively alleviate depressive and anxious symptoms among college students, with Baduanjin and Yijinjing showing superior intervention effects compared to Tai Chi.

Du et al. (2022) conducted a Meta-analysis on the effect of Tai Chi therapy on improving negative psychological symptoms such as anxiety and depression in college students, as well as the optimal intervention dosage. They found that Tai Chi can significantly reduce symptoms of depression and anxiety in college students, with an intervention period of more than 12 weeks and a frequency of three times a week being the most effective. Chen et al. (2015) compared the effects of Baduanjin intervention and relaxation training on college students and found that individuals in the Baduanjin intervention group showed significant improvements in depression scores compared to before the intervention, while there was no significant change in the assessment results before and after the intervention in the control group.

Yu et al. (2021) implemented an intervention program involving sequential practice of 14-form Tai Chi, 12-form health-preserving exercise, Baduanjin, and Wuqinxi among college students with mild depression. Compared to a control group without any intervention, the study showed significant reductions in SDS (Self-Rating Depression Scale), SAS (Self-Rating Anxiety Scale), and PSQI (Pittsburgh Sleep Quality Index) total scores (*p* < 0.05), indicating that TCM health-preserving exercises can improve depressive symptoms and sleep quality among college students. Gong (2024), through analyzing exercise prescriptions for depression among college students, pointed out that traditional exercises such as Tai Chi have satisfactory effects on improving depression and depressive states among college students. Research has found that Baduanjin can effectively relax the limbs, muscles, mind, and spirit of college students with depression, exhibiting emotional and psychological regulatory effects that can alleviate or even eliminate exercise barriers, thereby enhancing their physical functions and quality of life. Tai Chi can regulate both physiological and psychological aspects, ultimately achieving the goal of treating depression (Gan and Zhong, 2023).

Yu et al. (2019) conducted a META analysis comparing Tai Chi intervention with conventional aerobic exercise intervention among college students with depressive tendencies or depression. The study found that practicing Tai Chi can effectively improve depression among college students, and compared to conventional aerobic exercise with a single format, college students showed longer duration and better persistence in Tai Chi training. Tan and Tan (2020) applied health qigong Baduanjin to intervene in college students with mild to moderate depression, resulting in reduced SDS scores, heart rate, and blood pressure, as well as decreased sensitivity of norepinephrine (NE) and 5-hydroxytryptamine (5-HT) receptors, thereby improving depression levels among college students. Zhang et al. (2023) randomly divided college students with anxiety and depression into an experimental group and a control group. The experimental group underwent a 5-week intervention using Tai Chi Eight Methods and Five Supplements, while the control group did not engage in any exercise intervention. The study found that the SAS and SDS scores of the experimental group were significantly lower than those of the control group (*p* < 0.01). Through RS-fMRI scanning, the fALFF values in the right middle frontal gyrus, orbital part (Frontal_Mid_Orb_R) of the experimental group were significantly higher (*p* < 0.05), while those in the left middle frontal gyrus (Frontal_Mid_L) were significantly lower (*p* = 0.001, *p* < 0.05) compared to the control group. This suggests that Tai Chi Eight Methods and Five Supplements may alleviate anxiety and depression among college students by regulating the activity of Frontal_Mid_L and Frontal_Mid_Orb_R.

As mentioned above, traditional Chinese medicine health preservation exercises constitute a broad conceptual category, encompassing Tai Chi, Baduanjin, Wuqinxi, etc. To clearly compare the differences among these experiments, particularly in terms of key information such as the number of participants, gender, age, experimental grouping, intervention frequency, and intervention duration, we have created Table 1 to provide a detailed statistical overview of the relevant experiments.

**Table 1:** Statistical analysis of experiments on the intervention of TCM health preservation exercises for depression among college students.

Format	Number	Author	Sample size	Male	Female	Age	Intervention frequency	Duration	Measurement tool	Grouping
Tai Chi	1	Hu (2017)	60	/	/	/	3 times/1 week	4 weeks	SDS	Mind-adjustment group (Tai Chi + mental imagery) Movement group (Tai Chi) Control group
Tai Chi	2	Li et al. (2017)	58	21	37	20–22	3 times/1 week	12 weeks	SDS	Experimental group (Tai Chi 4 times a week) Control group 1 (Tai Chi 3 times a week) Control group 2 (No intervention)
Tai Chi	3	Zhao (2018)	60	/	/	/	/	/	/	Experimental group (Tai Chi) Control group (Basketball)
Tai Chi	4	Bo (2008)	64	/	64	18	1 time/1 week	14 weeks	CES-D, BDI	Experimental group (Tai Chi) Control group (Physical exercise)
Tai Chi	5	Xu et al. (2016)	108	38	70	18–22	2 times/1 week	6 weeks	SDS, HAMD	Treatment group (Tai Chi + accupoint application and pressure) Tai Chi group Control group
Wu Qin Xi	6	Tao and Pengyu (2017)	50	30	20	18–22	2 times/1 week	10 weeks	SCL-90	Male group (Wu Qin Xi) Female group (Wu Qin Xi)
Wu Qin Xi	7	Cheng et al. (2016)	30	/	/	/	/	/	/	Intervention group (Wu Qin Xi) Non-intervention group (No intervention)
Wu Qin Xi	8	Wang and Qin (2015)	60	28	32	21–24	3 times/1 week	12 weeks	DSM-IV	Intervention group (Wu Qin Xi) Control group
Wu Qin Xi	9	Qiu (2011)	60	/	/	18	7 times/1 week	8 weeks	HAMD, SDS, PSQI	Experimental group (Wu Qin Xi) Control group (Walking training)
Eight Sections of Brocade	10	Liu et al. (2008)	100	/	/	18–22	5 times/1 week	12 weeks	SCL-90	Experimental group (Ba Duan Jin) Control group (No intervention)
Eight Sections of Brocade	11	Su (2014)	222	/	/	18–22	5 times/1 week	12 weeks	Main outcome measures: SCL-90, CPSS, GSES; Secondary outcome measures: SES, POMS, WHOQOL-BREF, PSQI	Experimental group (Ba Duan Jin) Control group (No intervention)
Eight Sections of Brocade	12	Tan and Tan (2020)	70	/	/	/	/	/	/	Experimental group (Ba Duan Jin) Control group (No intervention)
Eight Sections of Brocade	13	Yao et al. (2024)	160	35	125	18–21	7 times/1 week	4 weeks	SAS, SDS, PSQI	Ba Duan Jin group Comprehensive group (Ba Duan Jin + Five Element Music) Five Element Music group Control group (No intervention)
Yi Jin Jing	14	Shen et al. (2018)	26	/	/	/	/	/	/	Experimental group (Yi Jin Jing)
Yi Jin Jing	15	Yangjun (2013)	72	/	/	18–22	4 times/1 week	8 weeks	HAMD, SDS	Experimental group (Yi Jin Jing)
Yi Jin Jing	16	Wang et al. (2011)	120	/	/	18–24	≥5 times/1 week	8 weeks	SAS\SDS	Treatment group (Yi Jin Jing) Control group (Acupuncture) Blank group (No intervention)

In summary, in terms of intervention forms, a few researchers have adopted multiple TCM health-preserving exercises for intervention, while more researchers have used only one type of TCM health-preserving exercise. The control groups in the above experiments also vary, with some control groups receiving no intervention at all, while others are required to engage in certain exercise training. The experimental results suggest that TCM health-preserving exercises have a certain positive effect and relatively good intervention outcome on intervening in depression among college students.

### 2.2 Intervention of traditional Chinese medicine’s five-element music

The concept of music as a means of health preservation and treatment can be traced back to over two thousand years ago. In traditional Chinese characters, the homology of the characters *yue* (樂, music), *yao* (藥, medicine), and *liao* (療, treatment) serves as indirect evidence of the inherent connection between music, medication, and therapeutic practices. Similar to pharmacological treatments, music also possesses the capability to regulate and heal the human body (Li and Wang, 2021). The Records of the Grand Historian (Shi Ji) in the section of Music Book (Yue Shu) records, “Music is able to stimulate the flow of blood, circulate the spirit, and harmonize the mind.” References to the Five-Element Music Therapy can be found in the Yellow Emperor’s Inner Classic (Huang Di Nei Jing), a text dating back over 2,000 years, which advocates the ideas of “Five Tones Healing Diseases,” “The Correspondence of Five Tones,” and “All illnesses originate from the imbalance of Qi (vital energy), and cease with the sound.” This classic also describes “listening to music” as a supreme level of preventive medicine, emphasizing the concept of “superior doctors preventing disease” (Zhao et al., 2022; Huang et al., 2023).

The Yellow Emperor’s Inner Classic associates the five tones with the five elements, five internal organs, five colors, and five emotions. As recorded in Spiritual Pivot – The Invading Guest (Ling Shu – Xie Ke), “The liver belongs to wood, its corresponding tone is jue (horn), and its associated emotion is anger; the heart belongs to fire, its corresponding tone is zhi (scale), and its associated emotion is joy; the spleen belongs to earth, its corresponding tone is gong (palace), and its associated emotion is thought; the lung belongs to metal, its corresponding tone is shang (commercial), and its associated emotion is sorrow; the kidney belongs to water, its corresponding tone is yu (water-drop), and its associated emotion is fear” (Zhang and Liao, 2020). Based on the characteristics of the five internal organs, it is inferred that the five tones of gong, shang, jue, zhi, and yu correspond to the physiological and pathological conditions of the spleen, lung, liver, heart, and kidney, respectively. This forms foundation of the traditional Chinese medicine’s Five-Element Music Therapy, which aims to regulate the “five emotions” by exerting specific effects on the “five internal organs,” thereby achieving therapeutic objectives (Yuan et al., 2021; Cao, 2022). Moreover, the Qing Dynasty physician Wu Shiji’s “A Collection of Medical Prescriptions” (Li Liao Pian Wen) also mentions, “For illnesses arising from the seven emotions, viewing flowers can relieve boredom, and listening to music can dispel sorrow, sometimes even more effectively than taking medication.”

Animal experiments have demonstrated that Five-Element Music can elevate the levels of serotonin (5-HT) in the serum of depressed mice, reduce the content of monoamine oxidase (MAO) in the hippocampus and malondialdehyde (MDA) in the liver, thereby improving depressive behaviors (Cheng et al., 2015). Clinical studies conducted by Chen (2018) have found that Five-Element Music Therapy can alleviate depressive symptoms by possibly reducing the levels of interleukin-1β (IL-1β), interleukin-2 (IL-2), and interleukin-6 (IL-6) in the peripheral blood of depressed patients, thus improving their immune function. Research by Bradt et al. (2021) suggests that musical interventions may alleviate anxiety, depression, and other psychological symptoms among cancer patients. Wang et al. (2020) observed that among medical students with depressive emotions, those who received musical interventions scored significantly lower on SDS (Self-Rating Depression Scale) and SCL-90 depression subscales compared to the non-intervention group (*p* < 0.05). Chen et al. (2015) conducted a study on nursing students in Taiwan suffering from depression, administering Five-Element Music Therapy. They discovered that after the intervention, the participants’ scores on the Beck Depression Inventory (BDI) and saliva cortisol levels significantly decreased. When compared to a control group without any intervention, the BDI scores of the intervention group were significantly lower (*p* < 0.05) (Chen et al., 2015).

Yao et al. (2024) conducted an intervention experiment on some university medical students suffering from depression. They divided the participants into four groups: a comprehensive group (where subjects received interventions of both Five-Element Music and Baduanjin), a Five-Element Music intervention group, a Baduanjin intervention group, and a control group. The experiment demonstrated that the comprehensive intervention group had a more significant effect on depression than using Five-Element Music or Baduanjin alone. Bin et al. (2014) used Five-Element Music to intervene with 50 depressed college students, where they listened to music twice a week for 30 min each time, over a period of 6 weeks. The experiment employed the Self-rating Depression Scale (SDS) and the Symptom Checklist-90 (SCL-90) for assessment, proving that traditional Chinese Five-Element Music has a positive impact on improving depressive moods among college students. Xiaolin et al. (2012) conducted an intervention experiment on 60 depressed college students, dividing them into a Five-Element Music group (30 people) and a control group (30 people). The results showed that the total scores of SCL-90 and UPI in the Five-Element Music group were significantly lower than those in the control group, with a significant difference (*p* < 0.01). The scores for obsession, interpersonal relationship, depression, anxiety, paranoia, and psychoticism factors in the SCL-90 of the Five-Element Music group significantly decreased compared to before the treatment (*p* < 0.01) (Table 2).

**Table 2:** Statistical analysis of experiments on the intervention of five elements music for depression among college students.

Number	Author	Sample size	Male	Female	Age	Intervention frequency	Duration	Measurement tool	Grouping
17	Yuan et al. (2021)	77	0	77	/	4 times/1 week	12 weeks	SDS, MBI	Control group (Five-Element Music) Experimental group (Five-Element Music + Baduanjin)
18	Cao (2022)	150	55	95	/	7 times/1 week	8 weeks	HAMD, AMA	Control group (Western medicine + Traditional Chinese medicine) Treatment group (Western medicine + Traditional Chinese medicine + Five-Element Music + Baduanjin)
19	Chen (2018)	120	60	60	/	7 times/1 week	8 weeks	HAMD	Experimental group 1 (drug treatment) Experimental group 2 (drug treatment + Five-Element Music) Experimental group 3 (Five-Element Music) Control group (no intervention)
20	Wang et al. (2020)	66	/	/	/	1 times/1 week	8 weeks	SDS, SCl-90	Experimental group (Five-Element Music) Control group (no intervention)
21	Chen et al. (2015)	71	2	69	16–20	2 times/1 week	10 weeks	DMSRIA	Experimental group (Five-Element Music) Control group (no intervention)
22	Yao et al. (2024)	160	35	125	18–21	7 times/1 week	4 weeks	SAS, SDS, PSQI	Ba Duan Jin group Comprehensive group (Ba Duan Jin + Five Element Music) Five Element Music group Control group (No intervention)
23	Bin et al. (2014)	50	20	30	19–24	2 times/1 week	6 weeks	SDS, SCl-90	Experimental group (Five-Element Music) Control group (no intervention)
24	Xiaolin et al. (2012)	60	25	35	18–22	1 times/1 week	30 weeks	UPI, SCL-90	Experimental group (Five-Element Music) Control group (no intervention)

### 2.3 Acupuncture treatment

Acupuncture is the general term for Chinese medical needling and moxibustion techniques. Needling involves inserting specially made metal needles into specific accupoints in the patient’s body, and using techniques such as twisting and lifting to achieve the purpose of treating diseases. Moxibustion involves burning mugwort wool and applying it to the skin surface at accupoints, utilizing thermal stimulation to treat diseases.

Acupuncture therapy for depression has become a green therapy accepted and advocated by contemporary mainstream medicine due to its remarkable efficacy in regulating the spirit, nourishing the mind, soothing emotions, and alleviating stress (Li et al., 2023). “Suwen: Six Elements and Great Treatise on the Right Discipline” suggests that acupuncture can be used to warm, unblock, and drain qi stagnation caused by depression.

Meta-analyses have shown that the effectiveness of acupuncture in treating depression is comparable to that of antidepressant medications, with fewer adverse reactions (Zhang et al., 2010). Cai et al. (2021) randomly divided 60 depressed university students into an acupuncture group (*n* = 30) and a medication group (*n* = 30). The former received acupuncture combined with moxibustion, while the latter was orally administered fluoxetine hydrochloride capsules. Both groups were treated for 8 weeks. Results showed that the total effective rate was 92.86% in the acupuncture group, superior to 81.48% in the medication group (*p* < 0.05). Additionally, HAMD-17 and SDS scores decreased more significantly in the acupuncture group (*p* < 0.05), with faster onset and fewer adverse reactions. Li and Ji (2022) applied cognitive-behavioral therapy combined with acupuncture in treating depressed university students, achieving a total effective rate of 88.57%, and reducing Hamilton Depression Rating Scale (HAMD) and Self-rating Depression Scale (SDS) scores. Some scholars (Yu et al., 2019) believe that adolescent depression is closely related to impaired function of the Shaoyang channel as a pivot. Based on the theory of “Shaoyang as the pivot,” treating adolescent depression should focus on the Shaoyang channel, using acupuncture to regulate and smooth the qi flow of the Shaoyang channel, thereby facilitating the pivot function. Acupuncture points such as Fengchi, Fengfu, Tianzhu, Wangu, Yifeng, and Tianyou were selected to achieve the therapeutic effects of unblocking the Shaoyang channel, regulating the pivot function, and soothing emotions. Li and Yan (2016) analyzed and concluded that intervention with both medication and acupuncture therapy for university student depression yields more stable and rapid therapeutic effects with lower recurrence rates compared to other treatments (Table 3).

**Table 3:** Statistical analysis of experiments on the intervention of acupuncture treatment for depression among college students.

Number	Author	Sample Size	Male	Female	Age	Intervention Frequency	Duration	Measurement Tool	Grouping
25	Cai et al. (2021)	55	22	33	/	7 times/1 week	8 weeks	HAMD-17、SDS	Acupuncture-Moxibustion Group Medication Group (Fluoxetine Hydrochloride Capsule)
26	Li and Ji (2022)	70	25	45	17–23	7 times/1 week	4 weeks	HAMD、SDS	Experimental group (Cognitive Behavioral Therapy + Acupuncture Treatment) Control group (Cognitive Behavioral Therapy)

## 3 TCM pharmacological interventions

In TCM, depression is categorized under terms such as “Baihe Disease,” “Zangzao,” and “Yuzheng” (Zhai et al., 2020). The fundamental pathophysiology involves liver Qi stagnation, spleen Qi deficiency, and heart Qi deficiency, leading to imbalances in Yin, Yang, Qi, and blood. Clinical treatment is often tailored based on different diagnostic patterns.

Xiaoyao San, composed of Chaihu (Bupleurum), Baishao (White Peony Root), Danggui (Angelica), Fuling (Poria), Baizhu (Atractylodes), Zhi Gan Cao (Honey-fried Licorice), Bohe (Mint), and Shengjiang (Fresh Ginger), originates from the Taiping Huimin Heji Ju Fang. This formula is effective in soothing the liver, relieving depression, nourishing the blood, and strengthening the spleen, making it a commonly used remedy for liver Qi stagnation and spleen deficiency in clinical depression treatment. Animal studies show that Xiaoyao San increases plasma norepinephrine and hippocampal monoamine neurotransmitter levels, enhances brain 5-HT1A receptor and BDNF expression, and exerts antidepressant effects through multiple pathways (Quan et al., 2020).

Si Ni San, composed of Chaihu, Zhishi (Immature Bitter Orange), Shaoyao (Peony Root), and Licorice, is recorded in the Shanghan Lun and is known for its liver-soothing, spleen-regulating, Qi-adjusting, and depression-relieving properties. It is revered as a fundamental formula for liver Qi stagnation (Ru et al., 2020). Pharmacological research reveals that Si Ni San can regulate synaptic plasticity, serotonin (5-HT), brain monoamine neurotransmitters, and immune inflammation, contributing to its antidepressant effects (Li et al., 2017; Shen et al., 2020; Cao et al., 2019). Due to the difficulties in implementing pharmacological interventions for depression among college students, there is a scarcity of clinical research literature on the prevention and treatment of depression in college students using traditional Chinese medicine. Zhang et al. (2023) observed that in 42 adolescents with liver Qi stagnation type depression, combining Si Ni San with fluoxetine capsules achieved a total effective rate of 95.12%. This combination elevated serum BDNF and 5-HT levels, and reduced HAMD scores, PSQI scores, and TCM syndrome scores for emotional depression, indicating effective alleviation of clinical symptoms, improvement in serum biochemical markers, and enhanced sleep quality, with high safety (Table 4).

**Table 4:** Statistical analysis of experiments on the intervention of TCM for depression among college students.

Number	Author	Sample size	Male	Female	Age	Intervention frequency	Duration	Measurement tool	Grouping
27	Zhai et al. (2020)	60	24	36	/	7 times/1 week	6 weeks	HAMD, TCM syndrome score	Experimental group (TCM formula for soothing the liver and promoting blood circulation) Control group (Fluoxetine Hydrochloride Capsules)
28	Cao et al. (2019)	96 rats (animal experiment)	/	/	/	/	/	/	Control group (control + ddH2O) Maternal separation + double distilled water group (MS + ddH2O) Maternal separation + fluoxetine group (MS + fluoxetine, 5 g/kg) Maternal separation + low-dose SNS group (MS + SNS -low dose, 2.5 g/kg) Maternal separation + medium-dose SNS group (MS + SNS -medium dose, 5 g/kg) Maternal separation + high-dose SNS group (MS + SNS -high dose, 10 g/kg)
29	Zhang et al. (2023)	84	48	33	15–18	7 times/1 week	6 weeks	HAMD, PSQI, TCM syndrome score	Control group (Fluoxetine Hydrochloride Capsules) Observation group (Fluoxetine Hydrochloride Capsules + Sini San, a TCM treatment)

In summary, TCM is an important part of Chinese cultural heritage and a product of Chinese wisdom. It has shown significant effects in intervening in college students’ depression. TCM health preservation techniques, which combine physical activity, breath regulation, and psychological adjustment, effectively improve depressive symptoms and promote overall health among students. Many university physical education courses now include traditional health preservation techniques, and students’ familiarity with these methods contributes to their effectiveness. Furthermore, Five-Element Music Therapy, with its long history and modern research support, is a simple and viable option for addressing depression in college students, despite its limited application in this group. Acupuncture and internal herbal medicine, while effective, require professional diagnosis and treatment, which can limit their widespread use among students. Therefore, broad application of non-pharmacological TCM therapies like Ba Duan Jin, Tai Chi, Yi Jin Jing, Five Animal Frolics, and Five-Element Music Therapy could provide early prevention and intervention for student depression, benefit their mental and physical health, and help preserve and promote traditional Chinese culture. These methods are worthy of promotion and application in universities.
